# Do Activities Performed within the Intra-Contrast Rest Interval Affect Neuromuscular Performance during Complex-Contrast Training Protocols?

**DOI:** 10.5114/jhk/184168

**Published:** 2024-04-15

**Authors:** Daniel Gutiérrez-Flores, Pedro E. Alcaraz, Patrick Cormier, Antonio Martínez-Serrano, Tomás T. Freitas

**Affiliations:** 1 UCAM Research Center for High Performance Sport, UCAM Universidad Católica de Murcia, Murcia, Spain.; 2 Facultad de Deporte, UCAM Universidad Católica de Murcia, Murcia, Spain.; 3 SCS—Strength & Conditioning Society, Murcia, Spain.; 4 Canadian Sport Institute Pacific, Victoria, BC, Canada.; 5 Exercise Science, Physical and Health Education, University of Victoria, Victoria, BC, Canada.; 6 NAR—Nucleus of High Performance in Sport, São Paulo, Brazil.

**Keywords:** postactivation performance enhancement, kinetic analysis, athletic performance, neuromuscular, mechanical power, range of motion

## Abstract

The aim of this study was to analyze the acute effects of including different exercises within the intra-contrast rest interval (ICRI) of a complex-contrast training (CCT) session. Seventeen recreationally active males completed three different CCT protocols. Programs consisted of a contrast pair combining a moderate-intensity conditioning activity (i.e., a back squat) with a lower-body high-velocity exercise (i.e., a vertical jump) and only differed in the activities performed during the ICRI: 1) passive recovery (CCT_PASS_); 2) a mobility exercise (CCT_MOB_); and 3) an upper-body high-intensity strength exercise (i.e., a bench press) (CCT_STR_). Countermovement jump and bench press throw metrics were evaluated at baseline and after each set during the workout. The rate of perceived exertion was recorded post-session. Non-significant differences in performance were found between CCT_PASS_, CCT_MOB_ and CCT_STR_ throughout the session. Significant declines (p < 0.05) were observed for CMJ peak power in the last 2–3 repetitions of each set, irrespective of the protocol. CCT_STR_ was perceived as more intense than CCT_PASS_ and CCT_MOB_ (p < 0.05). From a neuromuscular performance perspective, including activities during the ICRI (mobility drills or high-intensity strength exercises) may be a suitable strategy to optimize CCT prescription since the acute responses were similar to those found with passive rest periods. Finally, prescribing a lower number of repetitions per set is recommended to attenuate mechanical performance impairment during CCT protocols, irrespective of the activities completed within the ICRI.

## Introduction

Success in high-intensity intermittent sports (e.g., team-sports) depends largely on the athletes’ ability to express high neuromuscular output (Freitas et al., 2017; Suchomel et al., 2016). Scientific evidence indicates that well developed strength and power capabilities may: 1) be key factors differentiating between athletes from different performance levels (Argus et al., 2012; Cometti et al., 2001; Wisloff, 2004); 2) contribute to superior athletic performance (Suchomel et al., 2016); 3) result in a reduced risk of injury (Case et al., 2020; Malone et al., 2019); and 4) favor post-competition recovery (Johnston et al., 2015; Owen et al., 2015). For these reasons, improving strength and power production is a common objective of coaches and sport scientists (Cormier et al., 2020; Turner et al., 2021) and studies investigating different training strategies that allow for optimized adaptations and, in turn, performance are of great interest.

Within the many differing strength-power training methodologies that can be implemented, complex-contrast training (CCT), a method that consists of combining a low-velocity, high-load exercise (termed a conditioning activity [CA]) with a biomechanically similar higher-velocity, lower-load exercise (e.g., plyometrics) in the same workout (Cormier et al., 2022), has been proposed as a suitable strategy for improving strength- and power-related capabilities (Abdi et al., 2019 Bauer et al., 2019; Biel et al., 2023; Cormier et al., 2020; Freitas et al., 2017; Strońska et al., 2018). Accordingly, recent reviews of the literature indicate that CCT interventions may improve lower-body maximal dynamic strength (Bauer et al., 2019; Cormier et al., 2020), linear sprint velocity (Cormier et al., 2022; Freitas et al., 2017; Thapa et al., 2021), vertical jump height (Cormier et al., 2020; Freitas et al., 2017; Thapa et al., 2021), and change of direction performance (Cormier et al., 2020; Thapa et al., 2021).

The mechanisms underlying the abovementioned improvements in athletic performance following CCT are not yet clear with different hypotheses found in the literature (Cormier et al., 2022). Ebben (2002) argues that CAs increase motor neuron excitability and reflex potentiation (possibly creating optimal training conditions for subsequent neuromuscular power production) and Cormie et al. (2011) sustain that priming the nervous system (via a CA) may facilitate the activation of the muscles involved in the correct order and magnitude. On the other hand, the post-activation performance enhancement (PAPE) (Blazevich and Babault, 2019) phenomenon is suggested as the primary mechanism underpinning CCT (Cormier et al., 2022). In brief, PAPE is the term used to describe any (transient) increase in maximal strength-power-speed performance following a CA and is thought to be related to changes in muscle temperature, the accumulation of intracellular fluid, and increased muscle activation (including motivational aspects) (Blazevich and Babault, 2019; Cuenca-Fernández et al., 2017). However, the mechanisms of PAPE are not clear and it has been suggested that CAs may serve simply as a warm-up that leads to subsequent improvements in performance (Blazevich and Babault, 2019). Regardless, and irrespective of its underlying factors, CCT is considered an effective method and has been shown to be beneficial for improving athletic abilities (Chamera et al., 2023; Freitas et al., 2017; García-Pinillos et al., 2014; Mikoƚajec et al., 2017; Spieszny and Zubik, 2018).

Current evidence indicates that PAPE is observed to a greater extent after five minutes (Blazevich and Babault, 2019), and that intra-contrast rest intervals (ICRI) longer than two minutes seem to lead to superior adaptations following CCT (Freitas et al., 2017). This may preclude the implementation of this training methodology in scenarios where the time available for resistance training is limited, such as team-sports (Weldon et al., 2022). Moreover, in these contexts, anecdotal evidence indicates that players usually prefer dynamic workouts (i.e., with short passive recovery time) and, thus, long rest intervals in the weight room (e.g., > 3 min) may negatively affect athletes’ motivation towards resistance training. Hence, coaches need to find strategies to efficiently use the rest period between exercises without impairing performance and potential adaptations, while maintaining athletes’ engagement in the training session. In their study on the practical applications and the program design of CCT interventions, Lim and Barley (2016) suggested the possibility of including mobility or stability exercises within the ICRI. Those authors theorized that this would not interfere with subsequent performance and would allow making training more time-efficient. However, this hypothesis is yet to be fully explored as most CCT protocols utilize passive ICRIs (i.e., no exercises are performed) (Bauer et al., 2019; Cormier et al., 2020; Freitas et al., 2017) and only two studies (Trybulski et al., 2022; Urbański et al., 2023) investigated the acute effects of completing different exercises during the ICRI. Moreover, no previous research has analyzed whether including mobility exercises during the recovery period has any beneficial or detrimental effect on subsequent performance. This information would be extremely helpful for practitioners since a deeper understanding of this phenomenon might allow exercise professionals to optimize the CCT prescription.

Therefore, the aim of this study was to analyze, in a lower-body CCT protocol, the effects of including, within the ICRI: 1) a mobility exercise of the thoracic spine; and 2) a CA involving other muscle groups (i.e., upper-body) on the rating of perceived exertion (RPE), and vertical jump and bench press throw (BPT) performance. We hypothesized that the inclusion of a mobility exercise during the ICRI would not impair performance, whereas performing a high-intensity strength exercise of a different movement pattern would lead to performance declines throughout the session.

## Methods

### Participants

Considering an effect size (ES) of 0.3 for a possible difference in CMJ height with respect to baseline (based on previous research (Chen et al., 2023)), an a priori sample size estimation was performed on G-Power (G-Power 3.1.9.2, Dusseldorf, Germany; http://www.gpower.hhu.de/) with repeated-measures, within-between interaction ANOVA as the statistical test, an alpha level of 0.05 and a power (1−β) of 0.80, a number of measures of 15 and a correlation among repeated measures of 0.5. The sample size estimation indicated that a minimum of 12 participants would be necessary for each condition. Therefore, accounting for potential dropouts, seventeen male adults (age = 25.6 ± 2.8 years, body mass = 80.6 ± 6.2 kg; body height = 177.9 ± 7.7 cm; relative strength: back squat = 1.85 ± 0.21 kg·kg^−1^; bench press = 1.23 ± 0.17 kg·kg^−1^) were recruited and participated in the study. All participants were recreationally trained (with experience playing different team-sports) and had at least three years of resistance training experience. Participants were not considered for inclusion if they sustained any injury during the three months prior to the experimental procedures or if they presented any medical condition that could limit their performance. Before starting the investigation, all participants were informed of the details of the intervention and signed an informed consent form, approved by the Ethics Committee of the Universidad Católica de Murcia (protocol code: CE032206; date of approval: 25 March 2022). All procedures were conducted according to the Declaration of Helsinki.

### Design and Procedures

A repeated-measures, crossover, quasi-experimental design was used with an experimental period lasting three weeks. All participants completed a familiarization session in which the exercises that composed the CCT protocols (i.e., a back squat, a bench press, a countermovement jump [CMJ] and a BPT) were performed. This session also allowed the determination of the approximated loads to be used during the exercise protocols. In the two following weeks, participants completed, in randomized order, three exercise sessions (one for each of the CCT protocol detailed below) separated by at least 72 h. Prior to testing, participants performed a standardized comprehensive warm-up that included general exercises (i.e., 8 min cycling, 3–4 min of active stretching and mobility drills of the lower and upper extremities, 3 sets of 10 repetitions of body weight push-ups, squats, lunges and jumps) and specific exercises (i.e., 2 sets of 3–4 repetitions of the back squat and bench press exercises with submaximal loads, corresponding to 70–80% of the load determined in the familiarization session for each lift, moved as fast as possible in the concentric phase).

### Exercise Load Determination

The exercise sessions’ loads were determined on the familiarization day. After the standardized warm-up, back squat and bench press incremental load tests were performed on a Smith Machine, as described elsewhere (Ortega-Becerra et al., 2021). Regarding the back squat, participants descended at a controlled velocity until the thighs were parallel to the ground and held this position for ~1 s. Then, they were verbally encouraged to complete the concentric phase of the lift at maximal velocity. The initial load was 50 kg and 20% of each participant’s body mass was progressively added on each set until maximal intended velocity was ~0.6 m•s^−1^, determined with a linear position transducer (Vitruve®, Madrid, Spain) attached to the barbell, perpendicular to the ground. For the bench press, the barbell was lowered until nearly touching the chest (without actual contact to avoid rebounding). The initial load was set at 30 kg and 10% body mass was progressively added until velocity reached ~0.45 m•s^−1^. In both exercises, participants were instructed to perform 3 repetitions in each set moving the barbell as fast as possible in the concentric phase and 4 to 6 sets were completed before achieving the intended movement velocity. Rest between sets was 3 min. The 1-repetition maximum (1RM) of each exercise was estimated from barbell velocity of the heaviest load lifted using previously published equations (Pareja-Blanco et al., 2020).

### Testing

*Vertical jump*. The CMJ was performed on a portable force plate sampling at 1000 Hz (Kistler 9286BA, Kistler Group, Winterthur, Switzerland) following the protocol described elsewhere (Markovic and Mikulic, 2010). The depth of the countermovement was self-selected to avoid any alteration in jump coordination. Recovery time between each of the five jumps was also self-selected allowing for adequate rest based on the individual (~10 to 15 s). ForceDecks software (Vald Performance®, Brisbane, Australia) was used to collect jump output variables (i.e., jump height and peak power), jump strategy metrics (i.e., the modified reactive strength index [RSI_mod_,] as well as braking phase duration) (Bishop et al., 2022) and eccentric (ECC) phase variables (i.e., ECC peak velocity and peak power).

*Barbell velocity tracking*. Barbell velocity during the back squat, bench press and the BPT exercises was tracked with a linear position transducer (Vitruve®, Madrid, Spain). Mean propulsive velocity, for all repetitions of the back squat and the bench press, along with peak velocity (ICC = 0.982 [0.961;0.993]; CV = 1.05), for the BPT, were recorded during the workout.

*Rate of perceived exertion*. The RPE 10-point adapted Borg scale (Herman et al., 2006) was used and data were recorded following each session. Approximately 20 min after the end of the exercise protocol, participants were asked: “How intense was the workout?” and presented with the RPE table. This time frame was selected so that the exercises performed at the end of the session would not influence the RPE of the entire bout (Day et al., 2004).

### Exercise Protocols

On the CCT session days, upon arrival at the research center, participants completed the standardized warm-up after which three CMJs and three BPTs (with a load that allowed reaching a peak velocity of ~1.3 m·s^−1^) were performed. The highest values were later used as a baseline reference for the corresponding exercise session. All CCT protocols consisted of three sets of a contrast pair combining a moderate-intensity CA (i.e., three repetitions of a back squat performed at a velocity of ~0.6 m·s^−1^, corresponding to ~80% 1RM) and a lower-body high-velocity exercise (i.e., five repetitions of a CMJ). Following each set, 1 min after performing the CMJs, five repetitions of the BPT (with the load used for the baseline set) were performed to determine how incorporating different activities during the 2 min and 30 s ICRI affected the execution of an upper-body ballistic action. A passive rest of 2 min was allowed between sets.

**Figure 1:**
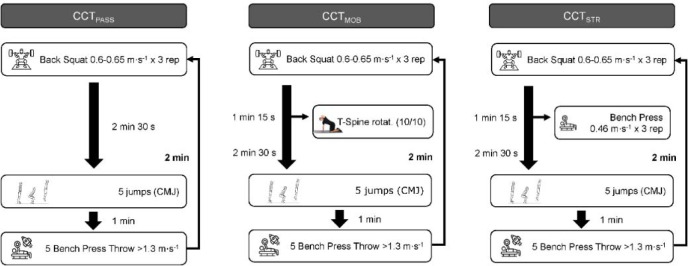
Schematic representation of the different CCT protocols.

All CCT protocols were identical except for the activities performed during the ICRI. In this regard, three different conditions were considered: 1) CCT with passive rest, as participants did not perform any exercise during the ICRI (CCT_PASS_); 2) CCT with a thoracic spine mobility exercise (i.e., thoracic spine rotation) during the rest interval (CCT_MOB_); and 3) CCT with a moderate- to high-intensity strength-oriented exercise of the upper-body (i.e., a bench press) during the ICRI (CCT_STR_). The representation and details of each protocol can be found in Figure 1.

### Statistical Analysis

Statistical analysis was performed using the software Jamovi® (v1.6.23). Data are presented as mean ± standard deviation (SD). The intraclass correlation coefficient (ICC) and the corresponding 95% confidence intervals (CI), as well as the coefficient of variation (CV), were used to verify the relative and absolute reliability, respectively. Normal distribution of the data was confirmed using the Shapiro-Wilk test. Differences in jump height, RSI_mod_, peak power, ECC peak velocity, ECC peak power, braking phase duration, BPT peak velocity and the RPE were determined using a linear mixed model test (with protocol, set and repetition as factors) with baseline values as covariates. When significant interactions were noted, pairwise comparisons were performed using Bonferroni’s post-hoc adjustments. A *p*-value of 0.05 was set for statistical significance. ESs were calculated using Cohen’s equations (Cohen, 1977) with the magnitude of standardized difference interpreted using the following thresholds: < 0.2, 0.2–0.6, 0.6–1.2, 1.2–2.0, 2.0–4.0, and > 4.0 for trivial, small, moderate, large, very large, and near perfect, respectively (Hopkins et al., 2009). To allow for inferences about the true values of the effect on the selected variables, 95% CIs were calculated.

## Results

A main effect of the protocol was found for CMJ height (*p* < 0.001; ICC = 0.972 [0.941;0.988]; CV = 1.85%), peak power (*p* < 0.001; ICC = 0.978 [0.954;0.991]; CV = 1.7%), RSI_mod_ (*p* = 0.005; ICC = 0.947 [0.891;0.977]; CV = 3.9%), braking phase duration (*p* = 0.007; ICC = 0.740 [0.538;0.879]; CV = 6.6%) and ECC peak velocity (*p* = 0.009; ICC = 0.847 [0.708;0.932]; CV = 5.2%), but not ECC peak power (*p* = 0.345; ICC = 0.900 [0.801;0.956]; CV = 8.5%). However, differences were non-meaningful as ESs were trivial (all ES < 0.2). A main effect of set was observed for all CMJ metrics (all *p* < 0.001) except braking phase duration (*p* = 0.092). Again, the magnitude of the differences was found to be trivial (all ES < 0.2). Regarding BPT peak velocity, main effects of protocol and set were found (all *p* < 0.001) with trivial ESs (all ES < 0.2). No protocol*set interaction was observed for any of the studied variables (*p*-values ranging from 0.494 to 0.976).

**Figure 2:**
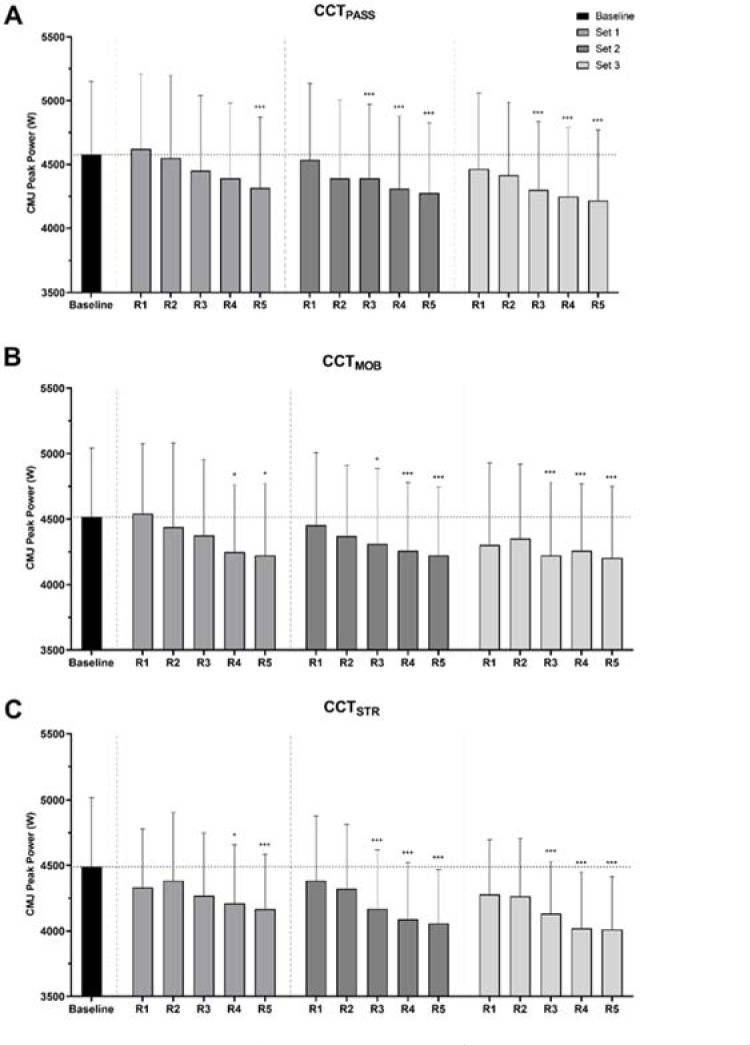
Countermovement jump peak power output from every repetition of each set in CCT_PASS_ (A), CCT_MOB_ (B) and CCT_STR_ (C). * p ≤ 0.05; *** p ≤ 0.001

Table 1 and 2 summarize the data obtained for the different CMJ and BPT variables, respectively, for every repetition of each set in CCT_PASS_, CCT_MOB_ and CCT_STR_. The only metric that yielded significant differences, with respect to baseline, was CMJ peak power. Significantly lower peak power was observed in the last repetition of the first set, and the last three repetitions of both the second and third sets in CCT_PASS_. Moreover, significant declines in CMJ peak power were found in repetitions 4 and 5 of the first set, and repetitions 3, 4 and 5 of the second and the third set in CCT_MOB_ and CCT_STR_ (Table 1 and Figure 2). No significant differences were found for any other variable, compared to baseline, irrespective of the CCT protocol.

**Table 1:** Descriptive data and statistical differences among all sets and repetitions for the multiple countermovement jump variables, considering the values of each CCT protocol.

	Countermovement Jump											
		**Height (cm)**		**Peak Power (W)**		**RSI_mod_** **(m·s^−1^)**		**Braking Phase (s)**		**ECC Peak Vel (m·s^−1^)**		**ECC Peak Power (W)**
**CCT_PASS_**	**Baseline**	40.3 ± 4.9		4576 ± 573		0.589 ± 0.115		0.295 ± 0.064		**−**1.42 ± 0.25		1780 ± 530
	**Set 1**											
	**Rep 1**	39.4 ± 4.5^T^		4622 ± 586^T^		0.597 ± 0.133^T^		0.286 ± 0.078^T^		**−**1.39 ± 0.26^T^		1794 ± 620^T^
	**Rep 2**	40.8 ± 4.6^T^		4549 ± 649^T^		0.615 ± 0.144^S^		0.282 ± 0.076^T^		**−**1.46 ± 0.23^T^		1948 ± 606^S^
	**Rep 3**	40.3 ± 4.3^T^		4450 ± 591^S^		0.586 ± 0.122^T^		0.299 ± 0.072^T^		**−**1.45 ± 0.26^T^		1880 ± 602^T^
	**Rep 4**	39.1 ± 4.5^S^		4391 ± 588^S^		0.568 ± 0.121^S^		0.293 ± 0.06^T^		**−**1.46 ±0.22^T^		1794 ± 537^T^
	**Rep 5**	39.1 ± 4.4^S^		4316 ± 554***^S^		0.563 ± 0.141^S^		0.304 ± 0.093^T^		**−**1.47 ± 0.29^T^		1995 ± 700^S^
	**Set 2**											
	**Rep 1**	39.8 ± 4.8^T^		4534 ± 602^T^		0.592 ± 0.117^T^		0.287 ± 0.066^T^		**−**1.41 ± 0.26^T^		1793 ± 561^T^
	**Rep 2**	39.3 ± 4.8^S^		4390 ± 613^S^		0.576 ± 0.12^T^		0.282 ± 0.052^S^		**−**1.49 ± 0.24^S^		1892 ± 545^S^
	**Rep 3**	39.1 ± 3.9^S^		4391 ± 580***^S^		0.566 ± 0.119^S^		0.291 ± 0.071^T^		**−**1.48 ± 0.26^S^		1892 ± 596^T^
	**Rep 4**	38.6 ± 4.4^S^		4309 ± 569***^S^		0.549 ± 0.11^S^		0.293 ± 0.052^T^		**−**1.49 ± 0.25^S^		1897 ± 597^S^
	**Rep 5**	39 ± 4.28^S^		4275 ± 551***^S^		0.563 ± 0.12^S^		0.289 ± 0.069^T^		**−**1.52 ± 0.26^S^		1993 ± 627^S^
	**Set 3**											
	**Rep 1**	39 ± 4.1^S^		4464 ± 595^T^		0.579 ± 0.113^T^		0.285 ± 0.067^T^		**−**1.41 ± 0.28^T^		1795 ± 615^T^
	**Rep 2**	39.1 ± 4.1^S^		4414 ± 573^S^		0.576 ± 0.103^T^		0.288 ± 0.06^T^		**−**1.47 ± 0.27^T^		1889 ± 593^T^
	**Rep 3**	38.2 ± 4.1^S^		4301 ± 533***^S^		0.56 ± 0.118^S^		0.282 ± 0.065^S^		**−**1.47 ± 0.27^T^		1912 ± 634^S^
	**Rep 4**	38.4 ± 4.6^S^		4249 ± 539***^S^		0.555 ± 0.107^S^		0.288 ± 0.061^T^		**−**1.49 ± 0.27^S^		1888 ± 599^T^
	**Rep 5**	38.2 ± 4.8^S^		4217 ± 553***^M^		0.536 ± 0.111^S^		0.301 ± 0.068^T^		**−**1.48 ± 0.25^S^		1878 ± 591^T^
	**Baseline**	40.3 ± 4.1		4515 ± 528		0.579 ± 0.105		0.304 ± 0.073		**−**1.42 ± 0.24		1751 ± 491
	**Set 1**											
	**Rep 1**	40.1 ± 3.6^T^		4543 ± 535^T^		0.584 ± 0.122^T^		0.288 ± 0.087^T^		**−**1.43 ± 0.24^T^		1803 ± 475^T^
**CCT_MOB_**	**Rep 2**	40.2 ± 3.9^T^	4439 ± 642^T^		0.584 ± 0.137^T^		0.302 ± 0.097^T^		**−**1.52 ± 0.27^S^		1951 ± 567^S^	
	**Rep 3**	39.8 ± 4.0^T^	4374 ± 583^S^		0.578 ± 0.127^T^		0.293 ± 0.071^T^		**−**1.52 ± 0.25^S^		1970 ± 578^S^	
	**Rep 4**	38.8 ± 4.4^S^	4249 ± 513*^S^		0.548 ± 0.121^S^		0.294 ± 0.07^T^		**−**1.49 ± 0.22^S^		1846 ± 481^T^	
	**Rep 5**	39 ± 3.8^S^	4222 ± 550*^S^		0.561 ± 0.114^T^		0.283 ± 0.068^S^		**−**1.55 ± 0.28^S^		2015 ± 625^S^	
	**Set 2**											
	**Rep 1**	40.2 ± 4.2^T^	4454 ± 555^T^		0.561 ± 0.129^T^		0.310 ± 0.098^T^		**−**1.44 ± 0.27^T^		1814 ± 566^T^	
	**Rep 2**	40.2 ± 4.0^T^	4370 ± 539^S^		0.581 ± 0.124^T^		0.290 ± 0.07^T^		**−**1.51 ± 0.25^S^		1906 ± 536^S^	
	**Rep 3**	39.8 ± 3.6^T^	4311 ± 575*^S^		0.576 ± 0.103^T^		0.289 ± 0.053^S^		**−**1.52 ± 0.24^S^		1921 ± 525^S^	
	**Rep 4**	39.8 ± 3.6^T^	4257 ± 522***^S^		0.561 ± 0.100^T^		0.300 ± 0.074^T^		**−**1.48 ± 0.26^S^		1820 ± 531^T^	
	**Rep 5**	39.3 ± 3.5^S^	4222 ± 525***^S^		0.551 ± 0.104^S^		0.295 ± 0.068^T^		**−**1.51 ± 0.25^S^		1865 ± 511^S^	
	**Set 3**											
	**Rep 1**	39.7 ± 4.8^T^	4302 ± 628^S^		0.579 ± 0.134^T^		0.290 ± 0.066^S^		-1.47±0.25^S^		1850 ± 506^T^	
	**Rep 2**	39.3 ± 4.4^S^	4351 ± 569^S^		0.575 ± 0.112^T^		0.284 ± 0.066^S^		-1.47±0.26^S^		1845 ± 540^T^	
	**Rep 3**	38.2 ± 5.0S	4222 ± 559***^S^		0.541 ± 0.121S		0.299 ± 0.068T		**−**1.62 ± 0.26S		1932 ± 571S	
	**Rep 4**	38.5 ± 4.4S	4257 ± 513***^S^		0.551 ± 0.108S		0.291 ± 0.068T		**−**1.44 ± 0.23T		1737 ± 476T	
	**Rep 5**	38.8 ± 4.7S	4203 ± 546***^S^		0.544 ± 0.114S		0.302 ± 0.075T		**−**1.44 ± 0.23T		1819 ± 581T	
	**Baseline**	39.7 ± 4.7	4489 ± 530		0.575 ± 0.11		0.289 ± 0.062		**−**1.42 ± 0.28		1765 ± 514	
**CCT_STR_**	**Set 1**											
	**Rep 1**	39.3 ± 4.9^T^	4331 ± 449^S^		0.571 ± 0.096^T^		0.284 ± 0.057^T^		**−**1.4 ± 0.26^T^		1745 ± 570^T^	
	**Rep 2**	39.2 ± 4.3^T^	4383 ± 522^S^		0.580 ± 0.114^T^		0.285 ± 0.062^T^		**−**1.47 ± 0.26^T^		1867 ± 528^T^	
	**Rep 3**	38.9 ± 4.3^T^	4268 ± 481^S^		0.564 ± 0.115^T^		0.297 ± 0.062^T^		**−**1.48 ± 0.27^S^		1855 ± 572^T^	
	**Rep 4**	38.3 ± 4.4^S^	4209 ± 449*^S^		0.549 ± 0.096^S^		0.289 ± 0.053^T^		**−**1.48 ± 0.26^S^		1799 ± 543^T^	
	**Rep 5**	38.5 ± 4.0^S^	4168 ± 418***^M^		0.547 ± 0.103^S^		0.295 ± 0.056^T^		**−**1.5 ± 0.26^S^		1920 ± 646^S^	
	**Set 2**											
	**Rep 1**	38.9 ± 4.4^T^	4384 ± 494^S^		0.568 ± 0.114^T^		0.295 ± 0.057^T^		**−**1.44 ± 0.28^T^		1787 ± 582^T^	
	**Rep 2**	39.3 ± 3.8^T^	4321 ± 493^S^		0.581 ± 0.113^T^		0.286 ± 0.054^T^		**−**1.51 ± 0.28^S^		1924 ± 594^S^	
	**Rep 3**	38.3 ± 4.2^S^	4169 ± 451***^M^		0.548 ± 0.094^S^		0.305 ± 0.066^S^		**−**1.48 ± 0.29^S^		1866 ± 666^T^	
	**Rep 4**	37.5 ± 4.0^S^	4089 ± 431***^M^		0.525 ± 0.105^S^		0.308 ± 0.06^S^		**−**1.45 ± 0.27^T^		1770 ± 578^T^	
	**Rep 5**	37.6 ± 3.3^S^	4057 ± 410***^M^		0.533 ± 0.101^S^		0.306 ± 0.072^S^		**−**1.46 ± 0.29^T^		1795 ± 563^T^	
	**Set 3**											
	**Rep 1**	38.4 ± 4.3^S^	4279 ± 419^S^		0.546 ± 0.096^S^		0.299 ± 0.061^T^		**−**1.44 ± 0.30^T^		1772 ± 634^T^	
	**Rep 2**	38.3 ± 3.7^S^	4265 ± 443S		0.552 ± 0.113 ^S^		0.297 ± 0.047^T^		**−**1.49 ± 0.26^S^		1834 ± 554^T^	
	**Rep 3**	38.1 ± 3.9^S^	4133 ± 396***^M^		0.536 ± 0.101 ^S^		0.314 ± 0.061^S^		**−**1.45 ± 0.26^T^		1709 ± 489^T^	
	**Rep 4**	36.9 ± 4.1^M^	4020 ± 430*** ^M^		0.514 ± 0.093 ^S^		0.302 ± 0.052^S^		**−**1.5 ± 0.26^S^		1806 ± 513^T^	
	**Rep 5**	37.2 ± 2.5^M^	4012 ± 403*** ^M^		0.512 ± 0.068**^M^**		0.308 ± 0.052^S^		**−**1.49 ± 0.28^S^		1806 ± 573^T^	

CCT_PASS_ = Complex-Contrast Training protocol with passive intra-contrast rest interval; CCT_MOB_ = Complex-Contrast Training protocol with the mobility exercise during the intra-contrast rest interval; CCT_STR_ = Complex-Contrast Training protocol with the strength exercise during the intra-contrast rest interval.

ECC = Eccentric; Rep = Repetition; RSI_mod_ = Modified Reactive Strength Index; Vel = Velocity.

* p ≤ 0.05; *** p ≤ 0.001 with respect to baseline values.^T^ trivial effect size with respect to baseline; ^S^ small effect size with respect to baseline; ^M^ moderate effect size with respect to baseline

**Table 2:** Descriptive data and statistical differences among all sets and repetitions for the bench press throw peak velocity, considering the values of each CCT protocol.

Bench Press Throw		
		**Peak Vel (m·s^−1^)**
**CCT_PASS_**	**Baseline**	2.15 ± 0.16
	**Set 1**	
	**Rep 1** **Rep 2** **Rep 3** **Rep 4** **Rep 5**	2.13 ± 0.14^T^ 2.14 ± 0.15^T^ 2.12 ± 0.16^T^ 2.1 ± 0.16^S^ 2.07 ± 0.15^S^
	**Set 2**	
	**Rep 1** **Rep 2** **Rep 3** **Rep 4** **Rep 5**	2.15 ± 0.16 ^T^ 2.15 ± 0.16^T^ 2.12 ± 0.17^T^ 2.1 ± 0.18^S^ 2.06 ± 0.18^S^
	**Set 3**	
	**Rep 1** **Rep 2** **Rep 3** **Rep 4** **Rep 5**	2.14 ± 0.16^T^ 2.14 ± 0.16^T^ 2.11 ± 0.18^S^ 2.08 ± 0.16^S^ 2.07 ± 0.18^S^
**CCT_MOB_**	**Baseline**	2.14 ± 0.15
	**Set 1**	
	**Rep 1** **Rep 2** **Rep 3** **Rep 4** **Rep 5**	2.17 ± 0.16^T^ 2.16 ± 0.15^T^ 2.12 ± 0.18^T^ 2.09 ± 0.18^S^ 2.07 ± 0.17^S^
	**Set 2**	
	**Rep 1** **Rep 2** **Rep 3** **Rep 4** **Rep 5**	2.16 ± 0.16^T^ 2.16 ± 0.16^T^ 2.12 ± 0.16^T^ 2.09 ± 0.17^S^ 2.05 ± 0.18^S^
	**Set 3**	
	**Rep 1** **Rep 2** **Rep 3** **Rep 4** **Rep 5**	2.16 ± 0.17^T^ 2.15 ± 0.16^T^ 2.11 ± 0.17^T^ 2.09 ± 0.17^S^ 2.07 ± 0.16^S^
**CCT_STR_**	**Baseline**	2.1 ± 0.13
	**Set 1**	
	**Rep 1** **Rep 2** **Rep 3** **Rep 4** **Rep 5**	2.12 ± 0.15^T^ 2.12 ± 0.16^T^ 2.08 ± 0.17^T^ 2.03 ± 0.16^S^ 2.02 ± 0.16^S^
	**Set 2**	
	**Rep 1** **Rep 2** **Rep 3** **Rep 4** **Rep 5**	2.11 ± 0.16^T^ 2.1 ± 0.16^T^ 2.07 ± 0.15^S^ 2.03 ± 0.17^S^ 2.02 ± 0.16^S^
	**Set 3**	
	**Rep 1** **Rep 2** **Rep 3** **Rep 4** **Rep 5**	2.09 ± 0.16^T^ 2.08 ± 0.15^T^ 2.05 ± 0.16^S^ 2.02 ± 0.16^S^ 2.01 ± 0.17^S^

CCT_PASS_ = Complex-Contrast Training protocol with passive intra-contrast rest interval; CCT_MOB_ = Complex-Contrast Training protocol with the mobility exercise during the intra-contrast rest interval; CCT_STR_ = Complex-Contrast Training protocol with the strength exercise during the intra-contrast rest interval. Vel = Velocity.

* p ≤ 0.05; *** p ≤ 0.001 with respect to baseline values.

T trivial effect size with respect to baseline; ^S^ small effect size with respect to baseline.

**Figure 3:**
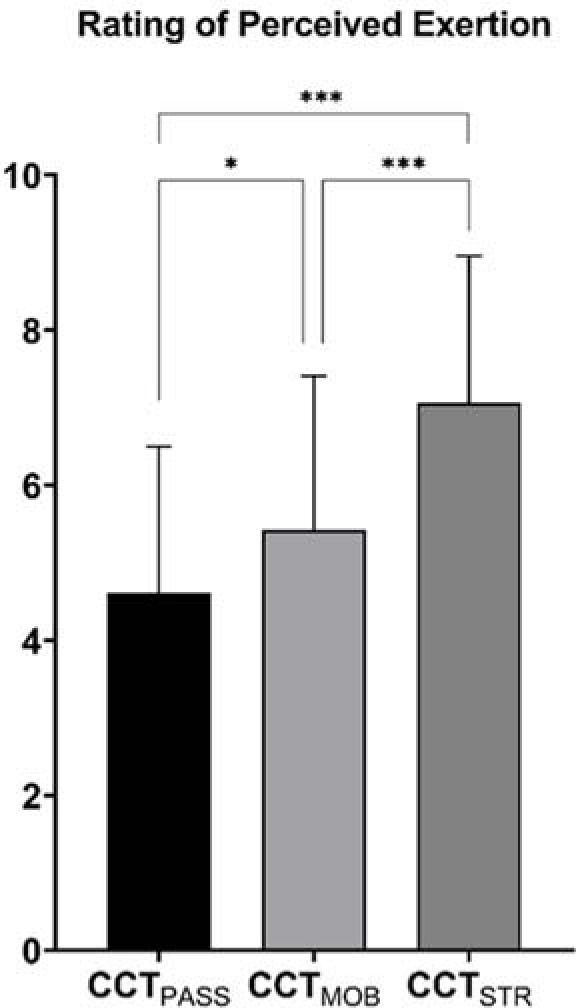
Rating of perceived exertion (RPE) reported after each CCT protocol. * p ≤ 0.05; *** p ≤ 0.001

Regarding RPE, significantly higher (*p* < 0.001) values were reported after CCT_STR_ with respect to both CCT_PASS_ (ES = 1.21 [0.47;1.94]) and CCT_MOB_ (ES = 0.75 [0.06;1.45]) and when comparing CCT_MOB_ and CCT_PASS_ (*p* = 0.022; ES = 0.45 [0.23;1.13]) (Figure 3).

## Discussion

The present study was designed to address a common concern of practitioners about how to efficiently prescribe CCT protocols using the resting time between the exercises of a complex-contrast pair to perform complementary exercises during CCT protocols. As such, the aim was to investigate the effects of including, within the ICRI, a mobility exercise or a high-intensity strength exercise of another muscle group on vertical jump and BPT performance. The main findings indicated that: 1) no PAPE was found, irrespective of the protocol performed; 2) active recovery periods (i.e., CCT_MOB_ or CCT_STR_) resulted in similar acute responses throughout the session when compared to passive rest (CCT_PASS_); 3) identical performance impairment, expressed as CMJ peak power declines in the last 2−3 repetitions of each set, was observed in CCT_PASS,_ CCT_MOB_ or CCT_STR_; and 4) CCT_STR_ was perceived as more intense than CCT_PASS_ and CCT_MOB_. From a practical perspective, the present results suggest that including activities during the ICRI may be a suitable strategy to optimize the CCT prescription since, overall, the acute responses were similar to those found with passive rest periods.

One of the findings of the present study was that no PAPE effect was found (i.e., no transient performance improvement) for any of the variables in any of the protocols. The fact that a proper and comprehensive warm-up that included aerobic work, dynamic stretching and mobility drills, body-weighted exercises, explosive actions (i.e., jumps) and progressive loading sets of the back squat exercise were performed may explain the results. As noted by Blazevich and Babault (2019), more often than not, PAPE responses are actually “warm-up effects”. This idea is supported by different studies (Marshall et al., 2019; Mina et al., 2018) that also reported no PAPE when an event-specific progressive warm-up was provided. For instance, Mina et al (2018) found no improvement in jump height after the completion of a set of three back squats with 85% 1RM that was preceded by a jump-specific warm-up. Another potential reason for the absence of PAPE responses here may be related to the duration of the ICRI. Previous research (Köklü et al., 2022; Yuan et al., 2023) reported performance enhancements following ICRIs greater than four minutes, which suggests that intervals slightly longer than the ones used may be necessary to induce PAPE.

In line with the present results, several studies (Comyns et al., 2006; Ebben and Blackard, 1997) that examined the effects of CCT protocols on subsequent explosive movements found no alterations in performance (positive nor negative) when passive rest periods were prescribed within contrast pairs. However, evidence is scarce regarding the effects of including another CA or a mobility exercise during ICRIs. Trybulski et al. (2022) evaluated the acute effects of incorporating a low-intensity exercise (i.e., a Swiss ball leg curl) within an upper-body strength-power potentiating complex (i.e., bench press + BPT) when compared to a passive ICRI. Those authors found similar PAPE responses in both conditions and concluded that performing body-weighted auxiliary exercises during the ICRI could be a viable option to optimize the CCT prescription from a time-efficiency perspective (Trybulski et al., 2022). Along the same lines, Urbański et al. (2023) demonstrated that the completion of an active ICRI (i.e., bench press exercise at 75% of 1RM) did not significantly impact lower-body PAPE responses (i.e., in contrast pairs consisting of a back squat and a hip thrust paired with a CMJ and a broad jump, respectively) in comparison with a condition in which participants rested seated. Thus, to some extent, previous literature (Trybulski et al., 2022; Urbański et al., 2023) supports the current findings in the sense that matching neuromuscular performances were found when completing a task during the ICRI or when simply allowing a passive recovery between exercises. However, direct comparisons should be made with caution since: 1) Trybulski et al. (2022) investigated upper-body contrast pairs (as opposed to lower-body in the present investigation); and 2) both mentioned studies (Trybulski et al., 2022; Urbański et al., 2023) prescribed ICRIs considerably longer than those used here (i.e., ~6 min vs. 2 min 30 s), which may limit their application in time-constrained scenarios (e.g., team-sport settings).

When analyzing each of the exercise sets in CCT_PASS,_ CCT_MOB_ and CCT_STR_, no protocol*set interaction was observed (*p* values ranging from 0.568 to 0.978), which indicates that completing a passive rest, a mobility exercise or a high-intensity strength exercise of the upper limbs during the ICRI induced similar responses throughout the whole session. Regarding the CCT_STR_, these findings can be explained by the fact that high-intensity muscular contractions during resistance training mainly generate localized peripheral fatigue and low central fatigue (Tornero-Aguilera et al., 2022; Zając et al., 2015). Therefore, since the activity performed during the ICRI under this condition (i.e., a bench press at ~0.45 m•s^−1^) involved different muscle groups than the subsequent exercise (i.e., a vertical jump), it is not surprising that lower-body mechanical power expression was not significantly different in comparison with a passive rest. Considering CCT_MOB_, the activity performed during the ICRI (i.e., a T-spine mobility drill) could be considered a low-intensity exercise (Larson-Meyer, 2016) that might not have been able to generate central or peripheral fatigue levels greater than CCT_PASS_. Taken together, these interesting findings could facilitate the implementation of CCT protocols in sport contexts where the time available for resistance training is limited, as coaches can program more time-efficient sessions utilizing the ICRI to prescribe exercises (e.g., mobility- or strength-oriented) that address players' individual needs without acutely impacting their performance.

Of note, a deeper analysis of each repetition of the exercise session considering the different vertical jump and BPT metrics yielded significant small-to-moderate declines (*p* < 0.05) in CMJ peak power in the last 2–3 repetitions of each set, irrespective of the protocol. Still, it is worth noting that the magnitude of the differences, with respect to baseline, was higher in CCT_STR_ (i.e., ESs ranging from −0.43 to −0.99, from −0.25 to −0.57 and from −0.31 to −0.62, in CCT_STR,_ CCT_MOB_ and CCT_PASS_, respectively). Hence, based on the present findings, it appears that performing a lower number of repetitions per set (i.e., three or less) during CCT could be a viable option to minimize and attenuate impairment in performance throughout the session. Indeed, shorter set configurations have been shown to result in lower declines in force, velocity and power, and lower neuromuscular fatigue and jump height impairment when compared to longer set configurations (Piqueras-Sanchiz et al., 2022), possibly due to a superior maintenance of intramuscular phosphocreatine stores and greater resynthesis of ATP (Gorostiaga et al., 2012; Tufano et al., 2017). It is important to consider, however, that over time, with chronic implementation of CCT interventions, individual athlete’s strength and power levels could be expected to increase above baseline (Cormier et al., 2020, 2022), which may improve the athlete’s ability to maintain power output and avoid decrements in performance throughout the exercise session. As such, further research evaluating the chronic effects of these acute protocols is of interest.

Another aspect worth noting was that CCT_STR_ was perceived as more intense than both CCT_PASS_ and CCT_MOB_ (*p* < 0.001), which is not surprising given the relationship between the RPE and resistance training intensity (Day et al., 2004). A protocol involving high-intensity exercises requires increased motor unit recruitment and firing frequency, which, in turn, may increase the perception of effort (Day et al., 2004). This is important to be taken into account by strength and conditioning practitioners as it points towards a higher internal load of CCT_STR_ when compared to CCT_PASS_ and CCT_MOB_. As such, CCT_STR_ should be used cautiously in contexts where recovery is paramount (e.g., in-season congested weeks).

The present study is limited by the fact that the sample comprised only male participants, and that very specific exercise intensities, set configurations, ICRI duration, mobility exercises and CAs were used which limits the extrapolation of the results (e.g., longer rest periods could have modified the study outcomes). Another limitation is that the actual mechanisms of PAPE (e.g., muscle temperature, motoneuron excitability or reflex potentiation) were not investigated, although this was not a main objective of the study due to its applied and practical perspective. A unique feature of the present study that is important to highlight is the utilization of barbell velocity as a means to determine exercise intensity during each session, thus ensuring that the CA load was individually adjusted every workout. Further investigations are needed to determine the acute effects of CCT protocols with a lower number of repetitions per set and a higher number of sets. Furthermore, studies that include other types of CAs (e.g., isometric-based) or set configurations (e.g., cluster sets) would be of interest, since little is known about their acute or short-term effects.

## Conclusions

The findings of this study can be useful for coaches to design their CCT sessions more effectively and efficiently. The present results indicate that, if ICRIs of 2 min 30 s are prescribed, the inclusion of a mobility drill or a high-intensity strength exercise of a different body part completed during the resting period may be expected to induce similar acute responses throughout the session to a passive rest. As such, practitioners may optimize the CCT prescription by including different exercises during the ICRI. However, CCT_STR_ should be used with caution in some specific sport contexts (e.g., in-season congested weeks) since this protocol was perceived as more intense. Another aspect worth considering is that prescribing three sets of five repetitions may not be the most appropriate approach if practitioners want to attenuate mechanical performance impairment during the entirety of a CCT session. Based on the data herein, declines in CMJ peak power may be expected in the last 2–3 repetitions of each set, irrespective of the protocol. In this regard, possible alternatives could be to perform a lower number of total sets or complete less repetitions per set (i.e., no more than three).
